# Dissociable circuits for visual shape learning in the young and aging human brain

**DOI:** 10.3389/fnhum.2013.00075

**Published:** 2013-03-27

**Authors:** Stephen D. Mayhew, Zoe Kourtzi

**Affiliations:** ^1^School of Psychology, University of BirminghamBirmingham, UK; ^2^Laboratory for Neuro- and Psychophysiology, K.U. LeuvenLeuven, Belgium

**Keywords:** fMRI, aging, learning, visual categorization, pattern classification

## Abstract

Recognizing objects in cluttered scenes is vital for successful interactions in our complex environments. Learning is known to play a key role in facilitating performance in a wide range of perceptual skills not only in young but also older adults. However, the neural mechanisms that support our ability to improve visual form recognition with training in older age remain largely unknown. Here, we combine behavioral and fMRI measurements to identify the brain circuits involved in the learning of global visual forms in the aging human brain. Our findings demonstrate the learning enhances perceptual sensitivity in the discrimination of visual forms similarly in both young and older adults. However, using fMRI we show that the neural circuits involved in visual form learning differ with age. Our results show that in young adults visual shape learning engages a network of occipitotemporal, parietal, and frontal regions that is known to be involved in perceptual decisions. In contrast, in older adults visual shape learning engages primarily parietal regions, suggesting a stronger role of attentionally-guided learning in older age. Interestingly, learning-dependent changes are maintained in higher occipitotemporal and posterior parietal regions, but not in frontal circuits, when observers perform a control task rather than engaging in a visual form discrimination task. Thus, learning may modulate read-out signals in posterior regions related to global form representations independent of the task, whereas task-dependent frontal activations may reflect changes in sensitivity with training in the context of perceptual decision making.

## Introduction

Recognizing objects in cluttered scenes relies on our ability to extract features from noisy sensory inputs and integrate them into global forms. Learning is known to play a key role in facilitating performance in a wide range of perceptual skills and optimizing visual form recognition in young adults (for reviews: Goldstone, 1998; Fine and Jacobs, 2002). In particular, previous work has shown that learning facilitates the detection and recognition of targets in clutter (Dosher and Lu, 1998; Goldstone, 1998; Schyns et al., 1998; Gold et al., 1999; Kovacs et al., 1999; Sigman and Gilbert, 2000; Gilbert et al., 2001; Brady and Kersten, 2003) by enhancing the integration of relevant features and their segmentation from noisy backgrounds.

Recently, we have shown that learning enhances the ability not only of young, but also older adults to discriminate global forms embedded in clutter (Kuai and Kourtzi, 2013), despite age-related decline of visual functions (Owsley, 2011). This is consistent with previous work showing that training enhances performance in older adults in a range of perceptual tasks; that is, brightness discrimination (Ratcliff et al., 2006), acuity (Fahle, 1993), texture discrimination (Andersen et al., 2010), and motion direction discrimination (Ball and Sekuler, 1986; Bower and Andersen, 2011) tasks. However, the neural mechanisms that support our ability to improve visual form recognition with training in older age remain largely unknown.

**Figure 1:**
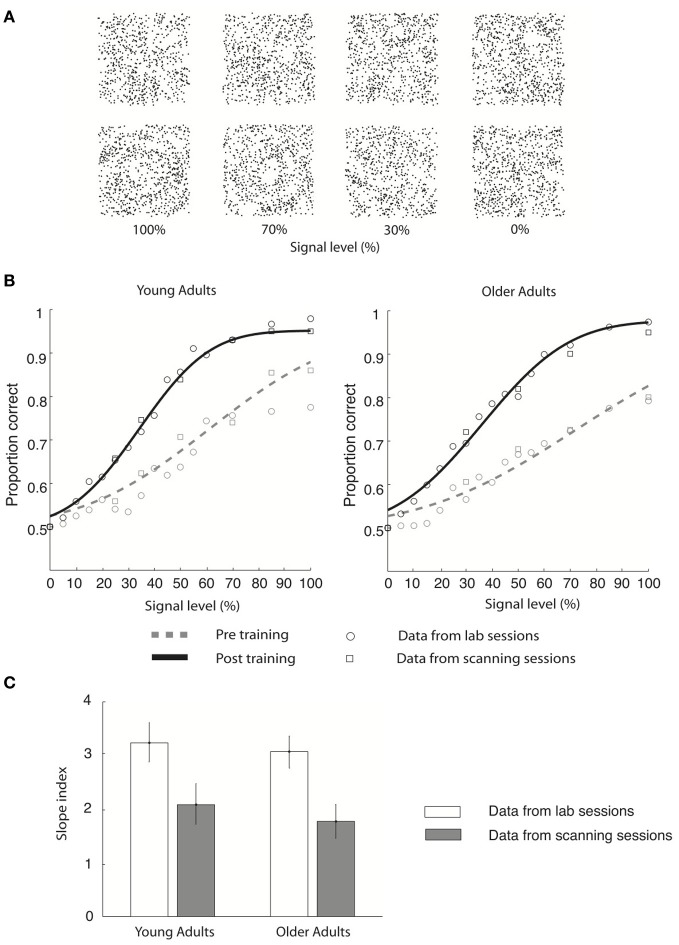
**Stimulus and behavioral data. (A)** Stimuli: four example Glass pattern stimuli at signal levels of 100, 70, 30, and 0% are shown for the radial category (upper row) and concentric category (lower row), respectively. **(B)** Behavioral data collected in the lab (circles) and the scanner (squares) are shown for young and older adults for both pre-training (gray dotted line) and post-training (black solid line) sessions. Only the cumulative Gaussian fits of the group averaged behavioral data from the lab are shown. **(C)** Group average slope index (difference between the slope of the psychometric functions before and after training) is plotted for behavioral data collected in the lab (gray) and during fMRI scanning (white) for both young and older adults. Error bars indicate the 95% confidence interval calculated using a bootstrap procedure.

Here, we combine behavioral and fMRI measurements to identify the brain circuits involved in the learning of global visual forms in young and older age. We used parametric manipulations of Glass patterns (Glass, 1969) that comprise oriented dot dipoles (Figure 1A). For these stimuli, small local changes to dot patterns have a predictable influence on the perception of global forms (Figure 1A: concentric vs. radial patterns). These stimuli are ideally suited for our purpose, as previous studies have shown that performance in discriminating global forms (before training) is similar in young and older adults (Habak et al., 2009). Thus, we were able to compare learning between age groups while avoiding potentially confounding performance differences. In particular, we trained observers to discriminate global form patterns (concentric vs. radial Glass patterns) that were embedded in parametrically manipulated background noise (Figure 1A). Our results showed similar behavioral learning effects for both young and older observers, suggesting that the ability for visual form learning is maintained in aging.

Using fMRI, we then asked which cortical regions mediate these learning-dependent improvements in visual form discrimination for young and older observers. Following our previous work (Li et al., 2009; Mayhew et al., 2012), we employed multi-voxel pattern analysis (MVPA) of fMRI signals (for reviews: Cox and Savoy, 2003; Haynes and Rees, 2006; Norman et al., 2006) to identify fine learning-dependent changes in neural preferences at the scale of large neuronal populations as revealed by fMRI. Using this methodology, we tested for regions that show learning-dependent changes in activation patterns corresponding to improved perceptual sensitivity in discriminating visual forms in cluttered backgrounds after training.

Previous brain imaging studies have shown that older adults exhibit different regional brain activity or recruitment of different brain regions compared to young adults (Grady et al., 1995; Cabeza et al., 1997), even when task performance is matched between age groups. Based on these findings and our previous work (Mayhew et al., 2010) we hypothesized that activation patterns related to visual form learning would be preserved in posterior areas but compromised in frontal brain circuits in older adults. To anticipate, our results show that in young adults visual shape learning engages a network of occipitotemporal, parietal, and frontal regions that is known to be involved in perceptual decisions (Newsome et al., 1989; Kim and Shadlen, 1999; Shadlen and Newsome, 2001; Heekeren et al., 2004, 2006). In contrast, in older adults we show that visual shape learning engages primarily parietal regions, suggesting a stronger role of attentionally-guided learning that enhances the perceptual salience of targets in noise (Gottlieb et al., 1998; Corbetta and Shulman, 2002; Roelfsema and van Ooyen, 2005; Mevorach et al., 2010).

## Materials and methods

### Participants

Two groups of ten observers (young adults: 6 males, 4 females, mean age: 21 ± 1.6 years; older adults: 3 males, 7 females, age 71 ± 2.6 years) participated in the main experiment. Two separate groups of eight observers each (young adults: 4 males, 4 females, age 22 ± 2.3; older adults: 4 males, 4 females, age 70 ± 3.1) participated in the control experiments. All observers had normal or corrected to normal vision and gave written informed consent. All observers in the older adult group completed a mini-mental state examination (MMSE) (Folstein et al., 1975) with scores within the range of normal cognitive ability [mean 28.7 (max score 30) ± 1.1]. The study was approved by the local ethics committee. The main experiment data recorded in the young adults was used in a previously published study but treated with different analysis methods (i.e., EEG-informed fMRI analysis) (Mayhew et al., 2012).

### Stimuli

We used Glass pattern stimuli (Glass, 1969) defined by white dot pairs (dipoles) displayed within a square aperture (7.7° × 7.7°) on a black background (100% contrast). The dot density was 3% and the Glass shift (i.e., the distance between two dots in a dipole) was 16.2 arc min. The size of each dot was 2.3 × 2.3 arc min^2^. These parameters were chosen based on pilot psychophysical studies and in accordance with previous studies (e.g., Wilson and Wilkinson, 1998; Li et al., 2009; Mayhew et al., 2012) showing that coherent form patterns are reliably perceived for these parameters. We generated radial (0° spiral angle) and concentric (90° spiral angle) Glass patterns by placing dipoles tangentially (concentric stimuli) or orthogonally (radial stimuli) to the circumference of a circle centered on the fixation dot. For each dot dipole, the spiral angle was defined as the angle between the dot dipole orientation and the radius from the center of the dipole to the center of the stimulus aperture. Each stimulus comprised signal dot dipoles that were aligned according to the specified spiral angle for a given stimulus, and noise dipoles for which the spiral angle was randomly selected. Stimuli were embedded in varying levels of noise by randomizing the orientation of a chosen percentage (0–100%) of dot dipoles (Figure 1A). Half of the observers were presented with clockwise spiral patterns (0° or 90° spiral angle), and half with anticlockwise spiral patterns (0° or −90° spiral angle). A new pattern was generated for each stimulus presented in a trial, resulting in stimuli that were locally jittered in their position.

To control for stimulus-specific training effects, and ensure generalization of learning, we used the following procedures. We trained observers using stimuli with Glass shift of 25 arc min, but tested (pre- and post-training test), and scanned, using stimuli with Glass shift of 30 arc min. Further, to control for local adaptation due to stimulus repetition, we generated different stimulus exemplars by randomly jittering (±5°) the spiral angle for each stimulus. These procedures ensured that learning could not be due to similar local cues between the stimuli used for training, tests, and scanning, but rather global features (i.e., spiral angle) used by the observers for stimulus discrimination.

### Design

All observers participated in two fMRI sessions. The first imaging session was preceded by a pre-training psychophysical test session (480 trials). The second imaging session was preceded by three sessions of psychophysical training outside the scanner comprising between five and eight runs (256 trials per run). At the end of this training, observers were tested on a post-training psychophysical test session (480 trials). All three training sessions were completed on consecutive days. The second scanning session was conducted on the following day after the post-training test session.

#### Psychophysical training

Psychophysical training was identical for both young and older adult observers in both the main and the control experiments.

First, observers were familiarized with the task and stimuli in a short practice session. Observers were shown 100% signal Glass patterns and were instructed to discriminate radial from concentric Glass patterns. Following this, two pre-training tests were performed where observers were presented with Glass patterns at 0, 5, 10, 15, 20, 25, 30, 35, 40, 45, 50, 60, 70, 85, 100% signal levels and were instructed to perform the same discrimination task. Sixteen stimuli were used for each signal level (8 radial, 8 concentric) totalling 240 stimuli per run. Stimuli were presented for 300 ms in a self-paced procedure without feedback. This pre-training test allowed us to assess each observer's initial discrimination performance before the first imaging session and training.

Following the first imaging session observers were presented with stimuli at 5, 10, 15, 20, 25, 30, 35, 40, 45, 50, 55, 60, 65, 70, 75% signal levels and were trained (self-paced procedure with audio error feedback) to discriminate between radial and concentric patterns. Each training session comprised multiple runs (ranging from 5 to 8 runs) with 256 trials per run. For each trial during training, the stimulus was presented for 300 ms. A white fixation square (7.7 × 7.7 arc min^2^) was presented at the center of each stimulus. Observers were instructed to indicate which category the stimulus belonged to by pressing one of two keys. Observers were trained until their performance reached a stable criterion level (80% correct twice on the training and 80% correct on the post-training test).

After training, observers were tested on 0, 5, 10, 15, 20, 25, 30, 35, 40, 45, 50, 55, 60, 65, 70, 75% signal levels in a post-training test (240 trials) during which stimuli were presented for 300 ms. To assess the result of training, no feedback was given during this post-training test.

#### fMRI measurements

For the main experiment, all observers participated in two scanning sessions during which they performed the discrimination task on the Glass pattern stimuli after training. For each observer, we collected data from 7 to 8 event-related runs in each session. The order of trials was matched for history (1 trial back) such that each trial was equally likely to be preceded by any of the conditions. The order of the trials differed across runs and observers. Both young and older adults were presented with 16 trials of each stimulus condition in each run (including one fixation condition during which only the fixation point was displayed at the center of the screen). For the young adults, each run comprised 129 trials (128 trials across all 8 conditions and one initial trial for balancing the history of the second trial) and two 9 s fixation periods (one in the beginning and one at the end of the run). For the older adults, each run comprised 97 stimuli (96 trials across conditions and one initial trial for balancing the history of the second trial). For the young adults the stimulus conditions comprised Glass patterns of 0° ± 1:5° or 90° ± 1:5° spiral angle at 0, 25, 35, 50, 70, 85, 100% signal level. For the older adults the stimulus conditions comprised Glass patterns of 0° ± 1:5° or 90° ± 1:5° spiral angle at 0, 30, 50, 70, 100% signal level. The difference in the number of conditions between young and older participants was due to the fact that older adults can typically stay still in the scanner for shorter time periods and may require more breaks than young adults. When choosing these stimulus conditions we sampled representative points on the psychometric functions for both young and older adults, while selecting a limited but adequate number of conditions to ensure that enough trials were recorded per condition and high quality signals were measured within the time constraints of fMRI scanning.

For both young and older adults, each trial lasted 3 s. For fixation trials, the fixation square was displayed for 3 s. For experimental trials (3 s long), each trial started with 200 ms stimulus presentation followed by 1300 ms delay during which a white fixation square was displayed at the center of the screen. The response procedure aimed to dissociate the motor response (button press) from the learned stimulus categories. All observers were familiarized with this procedure before scanning. The response procedure differed slightly between young and older adults. For the young adults, after this fixed delay, the fixation dot changed color to either green or red. This change in fixation color served as a cue for the motor response using one of two buttons. If the color cue was green, observers indicated concentric vs. radial by pressing the left vs. right finger/key, while if the color was red, the opposite keys were used (e.g., concentric = right key). The fixation color was changed back to white 300 ms before the next trial onset. For older adults, this procedure was modified to ensure that finger/key switching did not interfere with the ability of older adults to perform the task. After the 1300 ms fixed delay the color of the fixation dot changed to green. Here, this change in color provided a cue for the older adults to respond by pressing the left button for radial and the right for concentric. All older observers changed the hand that they used to respond with half way through the scanning session; the ordering of response hand was randomized across participants. The fixation color was changed back to white 300 ms before the next trial onset.

#### fMRI control experiment

For the control experiment, both young and older adult observers participated in two scanning sessions during which they performed a letter detection task. Observers did not perform the visual discrimination task during this experiment.

For each observer, we collected data from 7 to 8 event-related runs in each session. Glass patterns were presented in an event-related paradigm similar to that used in the main experiment. The order of the patterns differed across runs and observers but was matched for history (1 trial back) such that each trial was equally likely to be preceded by any of the conditions. Eight conditions (stimulus conditions of 0, 25, 35, 50, 70, 85, 100% signal level and fixation) with 16 trials per condition were presented in each run. Each run comprised 129 trials (128 trials across conditions and one initial trial for balancing the history of the second trial) and two 9 s fixation periods (one in the beginning and one at the end of the run).

A gray fixation dot (9 × 9 arc min^2^) was continually displayed at the center of the screen throughout each run. For fixation trials, only the fixation dot was displayed for 3 s. For experimental trials (3 s long), each trial started with 200 ms stimulus presentation followed by 1300 ms delay. At 500 ms intervals during both stimulus and fixation conditions, a capital black letter was displayed at the fixation point for 80 ms. At the start of each run a target letter was randomly selected from the Roman alphabet. Observers were informed of the target identity via the visual display and asked to respond with a button press at every presentation of the identified target letter. The target was presented with 15% probability, the other letters displayed in the sequence were chosen pseudo-randomly from the remaining Roman alphabet. A different target letter was used in each run, and the hand that observers used to respond to targets was balanced across subjects.

### Data acquisition

#### fMRI scanning

The experiments were conducted at the Birmingham University Imaging Center (3T Achieva scanner; Philips, Eindhoven, The Netherlands). EPI and T1-weighted anatomical (1 × 1 × 1 mm) data was collected with an eight channel SENSE head coil. EPI data (Gradient echo-pulse sequences) were acquired from 24 slices (whole brain coverage, TR: 1500 ms, TE: 35 ms, flip-angle: 73°, 2.5 × 2.5 × 4 mm resolution).

### Data analysis

#### Behavioral data analysis

Psychometric (proportion concentric) data were collected both in the lab and the MRI scanner separately for pre- and post-training sessions. The data were averaged across observers and fitted with a cumulative Gaussian function (Figure 1). The goodness of fit was assessed based on the significance of the correlation between the data and the cumulative Gaussian function using a maximum-likelihood method (Wichmann and Hill, 2001). Confidence intervals were calculated on the fits from 2000 bootstrap iterations of the data. Using this procedure for each individual observer's behavioral data, we identified the threshold (i.e., signal level at 78% correct) and slope of the psychometric function before and after training for each observer. The slope index (expressed in % signal level) was defined as the difference between the slope of the psychometric functions before and after training. A positive slope index indicates enhanced sensitivity after training.

#### fMRI data processing

MRI data was processed using Brain Voyager QX (Brain Innovations, Maastricht, The Netherlands). Anatomical data was used for 3D cortex reconstruction, inflation, and flattening. Pre-processing of functional data included slice-scan time correction, head movement correction, temporal high-pass filtering (3 cycles), and removal of linear trends. Trials with head motion larger than 1 mm of translation or 1° of rotation were excluded from the analysis. Spatial smoothing (Gaussian filter; full-width at half maximum, 6 mm) was performed only for group random effect analysis but not for data used for the multi-voxel pattern classification analysis. The functional images were aligned to anatomical data and the complete data were transformed into Talairach space. For each observer, the functional imaging data between the two sessions were co-aligned registering all volumes of each observer to the first functional volume of the first run and session. This procedure ensured a cautious registration across sessions. To avoid confounds from any remaining registration errors we compared fMRI signals between stimulus conditions within each session rather than across sessions. A gray-matter mask was generated for each observer in Talairach space from the anatomical data for selecting only gray-matter voxels for further analyses.

#### Multivariate brain mapping based on stimulus category

To test which cortical areas contain information that allows us to discriminate between stimulus categories (radial vs. concentric), we performed a multi-voxel searchlight analysis (Kriegeskorte et al., 2006). For each observer in the main experiment we pooled the data across scanning sessions. We defined a spherical aperture with radius of 9 mm and moved this aperture serially across gray-matter voxels in the whole cortex. For voxels within the aperture (98 voxels per aperture on average), we used a linear Support Vector Machine (SVM) (Vapnik, 1995) pattern classifier to classify fMRI signals based on stimulus category (radial vs. concentric). That is, we trained the classifier to associate the fMRI signal from each stimulus with a label (radial vs. concentric) that was determined by the category of the presented condition and tested the classifier's prediction on an independent data set.

We averaged both volumes from each trial (each MRI volume was acquired in 1.5 s and each trial lasted 3 s) to generate a single training pattern. To account for the hemodynamic delay, we shifted the fMRI time series by 3 volumes (4.5 s). To ensure generalization of the classification, we used a leave-one-run-out cross-validation procedure. For each cross-validation, one run was left out as an independent test dataset and the data from the rest of the runs was used as the training set. The classification accuracy for each aperture was obtained by averaging the prediction accuracy across cross-validations. The accuracy value for each voxel was obtained by averaging the accuracy values from all apertures in which this voxel was included (i.e., similar to smoothing the results using a sphere as a Kernel). To identify voxels with significantly higher accuracy than chance (50% correct) across observers and sessions we conducted a second level statistical analysis (*t*-test, *p* < 0.05, cluster threshold estimation 5 mm^2^).

#### fMR-metric functions based on multivoxel pattern analysis

We defined regions of interest (ROI) separately for young and older adults based on the searchlight analysis (**Table S1** for Talairach co-ordinates for all second-level significant activation clusters). Further, using standard retinotopic mapping procedures we identified V1 in each individual observer as a control visual area that is known to be engaged in the processing of basic visual features (e.g., orientation). To facilitate comparison between the main and control experiments, we identified ROIs for the control experiment based on the areas (localized by Talairach co-ordinates) from the main experiment, for both young and older adults.

To test which brain regions showed learning-dependent changes, we conducted MVPA on the activation patterns in these ROIs. For each observer, we selected voxels in each ROI that were significantly activated during all stimulus conditions compared to fixation (*p* < 0.05, uncorrected). We ordered these voxels by their *t*-value and selected up to 100 for each ROI and observer for further analysis. Prediction accuracy was found to saturate at this pattern-size across areas, resulting in a dimensionality compatible with previous studies (Haynes and Rees, 2005; Kamitani and Tong, 2005; Li et al., 2007). As the ROI were defined by pooling data across both the pre- and post-training sessions, a common set of voxels was selected for pattern classification of each session. Cautious alignment of the functional data across sessions ensured that the 100 voxels selected for MVPA were the same across sessions. Each voxel time course was z-score normalized for each experimental run separately. The data pattern for each trial was generated by shifting the fMRI time series by 3 volumes (4.5 s) to account for the hemodynamic delay.

Finally, we used a linear SVM and a leave-one-run-out cross-validation procedure for the pattern classification. We trained the classifier to associate fMRI signals across conditions (i.e., signal levels) with a label (radial vs. concentric). We averaged the two volumes from each trial (trial duration = 3 s, *TR* = 1.5 s) to generate one training pattern per trial. We then tested whether the classifier predicted the stimulus condition (radial vs. concentric) using an independent dataset. To ensure generalization of the classification, we used a leave-one-run-out cross-validation procedure. That is, for each cross-validation we left one run out as an independent test dataset. Data from the rest of the runs were used as the training set. It is important to note that the classification comparisons were independent from the voxel selection procedure. The voxel selection was conducted using only the training dataset (excluding the test dataset for each cross-validation).

For each observer, we calculated the mean performance of the classifier (proportion of trials classified correctly for each stimulus condition) in predicting whether each stimulus was radial or concentric across cross-validations. For each session, we averaged the classifier performance across observers and fitted the data using a cumulative Gaussian, similar to the behavioral data. We refer to these functions as fMR-metric functions as their estimation closely resembles that of the psychometric functions (Li et al., 2009). We measured the slope of the fMR-metric function for each session and observer. The slope index, the difference between the slope between sessions, was calculated and averaged across observers.

#### Eye movement analysis

We recorded eye-movements during the main experiment in the scanner from seven young and four older participants. Eye movements were recorded using the ASL 6000 Eye-tracker (Applied Science Laboratories, Bedford, MA) with 60 Hz temporal resolution. Eye-tracking data were pre-processed using the Eyenal software (Applied Science Laboratories, Bedford, MA) and analyzed using custom Matlab (Mathworks, MA) software. For each stimulus condition we computed horizontal eye position, vertical eye position, proportion of saccades at different saccade amplitude ranges, and number of saccades per trial, per condition.

## Results

### Behavioral performance: learning-dependent changes in perceptual sensitivity

We tested young and older observers' ability to discriminate global form patterns (i.e., radial vs. concentric) in noise (Figure 1A) and plotted their performance (proportion correct) as a function of stimulus signal level (psychometric function). Our results showed that behavioral sensitivity was enhanced after compared to before training for both young and older adults (Figure 1).

In particular, young adults reached the 78% performance threshold at 71.2% (±12.3%) signal before training, while at 40.6% (±6.3%) signal after training (Figure 1B). Specifically, the slope of the psychometric functions was steeper after (3.2 ± 0.2) compared to before (2.1 ± 0.3) training. Similar effects were observed in the older adults: perceptual sensitivity increased from 81.1% signal (±16.5%) to 43.6% signal level (±8.2%) at the 78% performance threshold (Figure 1B), resulting in a steeper slope after (3.0 ± 0.3) than before (1.8 ± 0.3) training. Similar effects were observed in behavioral data recorded during the scanning sessions (young adults: pre-training: 73.1 ± 13.5% signal; post-training: 41.5 ± 7.2% signal; older adults: pre-training: 79.8 ± 12.4% signal; post-training: 42.9 ± 7.7% signal). It is important to note that we did not observe any significant changes in the observer's criterion after training. In particular, for each observer we calculated percentage concentric responses for the 0 and 100% signal level conditions and computed the difference between each observer's performance and 50% chance performance both before and after training. A Two-Way ANOVA [factors: session (pre-, post-training) signal level (0%, 100%)] conducted on the percentage concentric response of the lab behavioral data found no significant effect of session for either young [*F*_(1, 18)_ = 0.25, *p* = 0.82] or older [*F*_(1, 18)_ = 0.42, *p* = 0.75] adults. These data suggest that the training procedure had successfully modified the observers' perceptual sensitivity rather than criterion.

To quantify learning-dependent improvement in perceptual sensitivity, we computed the difference in the slope of the psychometric functions between the pre- and post-training sessions for each participant (Figure 1C). A Three-Way ANOVA [Greenhouse-Geisser corrected, factors: session (pre-training, post-training), recording (lab, scanner), and age (young, old)] on the slope measurements showed a significant main effect of session [*F*_(1, 18)_ = 45, *p* < 0.0001], supporting training-dependent improvement in task performance (i.e., steeper slope measurements) across age groups. No significant main effect of either recording [*F*_(1, 18)_ = 0.26, *p* = 0.69] or age [*F*_(1, 18)_ = 2.7, *p* = 0.15] was found. The lack of significant interactions between session and recording [*F*_(1, 18)_ = 0.24, *p* = 0.88] suggests that similar learning effects were measured in the laboratory and fMRI scanner. The lack of a significant interaction between age and session [*F*_(1, 18)_ = 0.51, *p* = 0.49] and between age and recording [*F*_(1, 18)_ = 0.18, *p* = 0.67] suggests similar learning effects between age groups. Interestingly, no significant differences in slope were observed between young and older adults before [*t*_(1, 9)_ = 0.3, *p* = 0.67] or after [*t*_(1, 9)_ = 0.15, *p* = 0.89] training, allowing us to compare learning improvement between age groups while avoiding potentially confounding pre-training differences in performance.

### fMRI data: pattern classification across the whole brain

**Figure 2:**
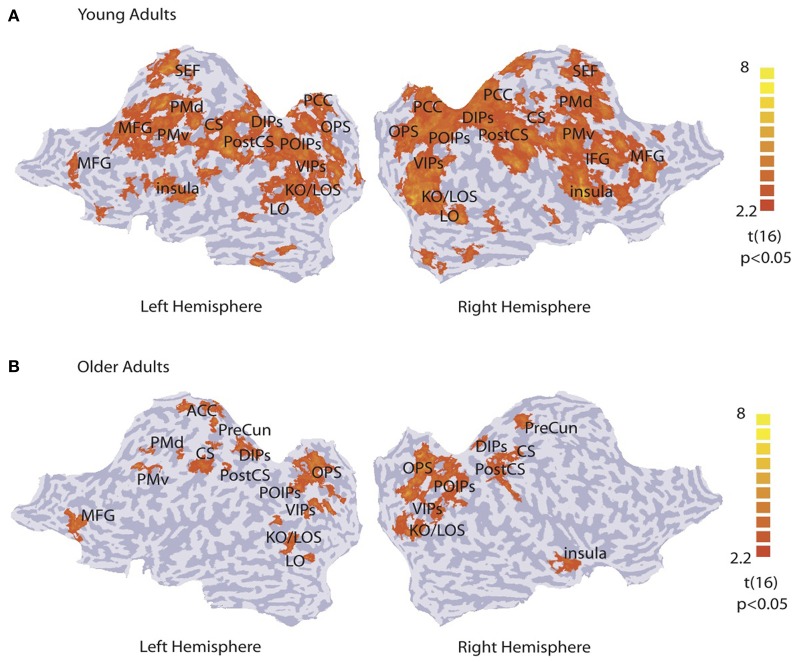
**Searchlight classification based on stimulus category.** *t*-statistic searchlight maps showing brain areas where classification of radial vs. concentric stimuli was significantly higher than chance level for **(A)** young and **(B)** older observers (data grouped across observers and sessions). Data are superimposed on flattened cortical surfaces of both hemispheres. Sulci are shown in dark gray, and gyri in light gray (**Table S1** for ROI definition and Talairach coordinates).

We identified activation patterns that discriminate between concentric and radial patterns by performing a multi-voxel searchlight analysis across the whole brain. Figure 2 shows group activation patterns averaged across both sessions (*p* < 0.05, cluster threshold estimation 5 mm^2^) for young (Figure 2A) and older (Figure 2B) adults. For the young adults, classification accuracy was significantly higher than chance in kinetic occipital (KO/LOS) and lateral occipital (LO) areas, cuneus and precuneus regions of the occipital parietal sulcus (OPS), parietal regions along the ventral, posterior and dorsal intraparietal sulcus (VIPs, POIPs, DIPS), motor regions comprising the post-central sulcus (PostCS), central sulcus (CS) and dorsal and ventral premotor regions (PMd, PMv), and frontal regions including the posterior cingulate cortex (PCC), insula, inferior frontal gyrus (IFG), medial frontal gyrus (MFG), and supplementary eye-field (SEF). For the older adults, classification accuracy was significantly higher than chance in occipitotemporal areas (KO/LOS, LO), cuneus and precuneus regions (OPS, PreCun), parietal regions along the intraparietal sulcus (VIPs, POIPs, DIPs), motor regions (PostCS, CS, PMd, PMv), and frontal regions including the left anterior cingulate cortex (ACC), right insula, and left MFG. These pattern classification analyses show that in both young and older adults, occipitotemporal, and posterior parietal regions contain information that allows us to discriminate between stimulus categories. However, informative activation patterns in frontal regions were predominantly evident in young rather than older adults.

In contrast, a conventional univariate GLM analysis did not reveal any significant difference in fMRI response between concentric vs. radial stimuli presented at 100% signal (data grouped across sessions, random effects, *p* < 0.05; cluster threshold estimation 5 mm^2^). Further, ROI-based analysis comparing the functional signal change between radial and concentric stimuli for each session showed no significant differences between the BOLD response to radial or concentric stimuli for either the pre-training or post-training session. In particular, a Three-Way repeated-measures ANOVA (ROI × session × stimulus) showed no significant effect of session {young: [*F*_(1, 19)_ = 0.25, *p* = 0.76]; older: [*F*_(1, 19)_ = 0.29, *p* = 0.73]} or stimulus {young: [*F*_(1, 19)_ = 0.75, *p* = 0.45]; older: [*F*_(1, 19)_ = 0.18, *p* = 0.82]} and no significant interaction between session and stimulus {young: [*F*_(1, 9)_ = 0.15, *p* = 0.95]; older: [*F*_(1, 19)_ = 0.84, *p* = 0.37]}. These findings are consistent with previous work showing that multivariate methods that pool weak activation biases across voxel patterns are more sensitive than conventional methods in discerning signals related to different stimulus categories and revealing learning-dependent changes in the discrimination of visual forms (Li et al., 2009).

### fMR-metric functions for young and older participants

The searchlight multivariate analysis showed that occipitotemporal, parietal, and frontal brain regions contain information that discriminates between visual categories (Figure 2). We subsequently generated fMR-metric functions (Li et al., 2009) to investigate which of these cortical regions show changes in fMRI activation patterns that relate to behavioral improvement in observers' sensitivity after training.

**Figure 3:**
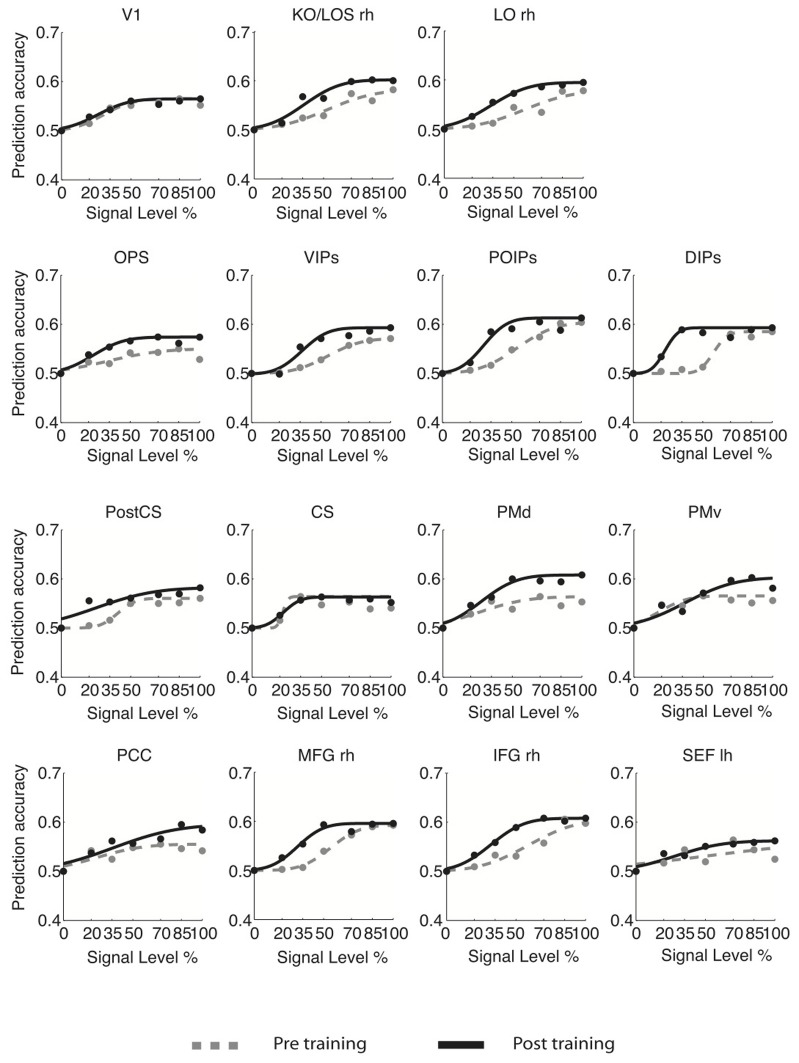
**fMR-metric functions for young adults.** fMR-metric functions are shown for each ROI, for both pre- (gray dotted lines) and post-training (black solid lines) sessions. Plotted data points depict the classifier predictions at each stimulus condition, averaged across observers. Only ROIs with significant fits (**Table S2**) for both sessions are shown.

**Figure 4:**
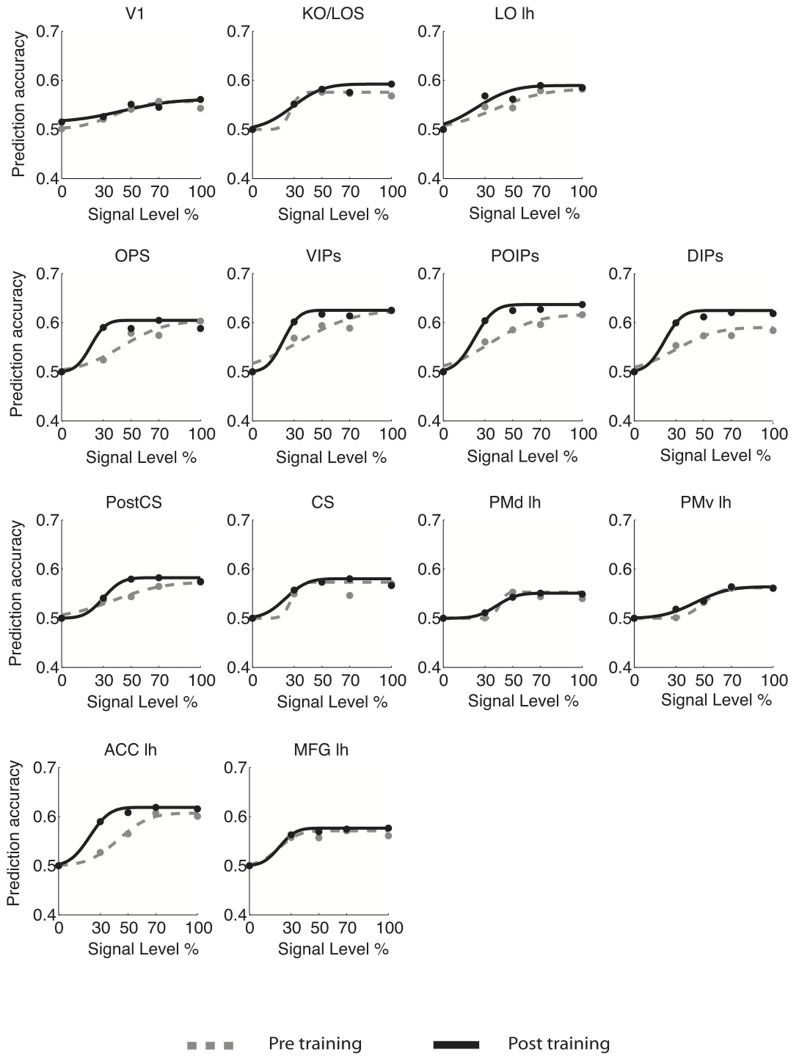
**fMR-metric functions for older adults.** fMR-metric functions are shown for each ROI, for both pre- (gray dotted lines) and post-training (black solid lines) sessions. Plotted data points depict the classifier predictions at each condition, averaged across observers. Only ROIs with significant fits (**Table S2**) for both sessions are shown.

For young adults (Figure 3), the slope of the fMR-metric functions increased after training in occipitotemporal (LO), parietal (VIPs, POIPs, DIPs, OPS), the PostCS, PMv, PMd, and frontal (PCC, MFG, IFG) areas. For older adults (Figure 4), steeper slopes after training were observed for the fMR-metric functions in parietal areas (VIPs, POIPs, DIPs, OPS), PostCS, and the anterior cingulate (ACC). These findings are consistent with the behavioral results showing improvement in perceptual sensitivity (i.e., steeper slopes of the psychometric functions) following training, and suggest training-induced brain changes for both young and older adults.

**Figure 5:**
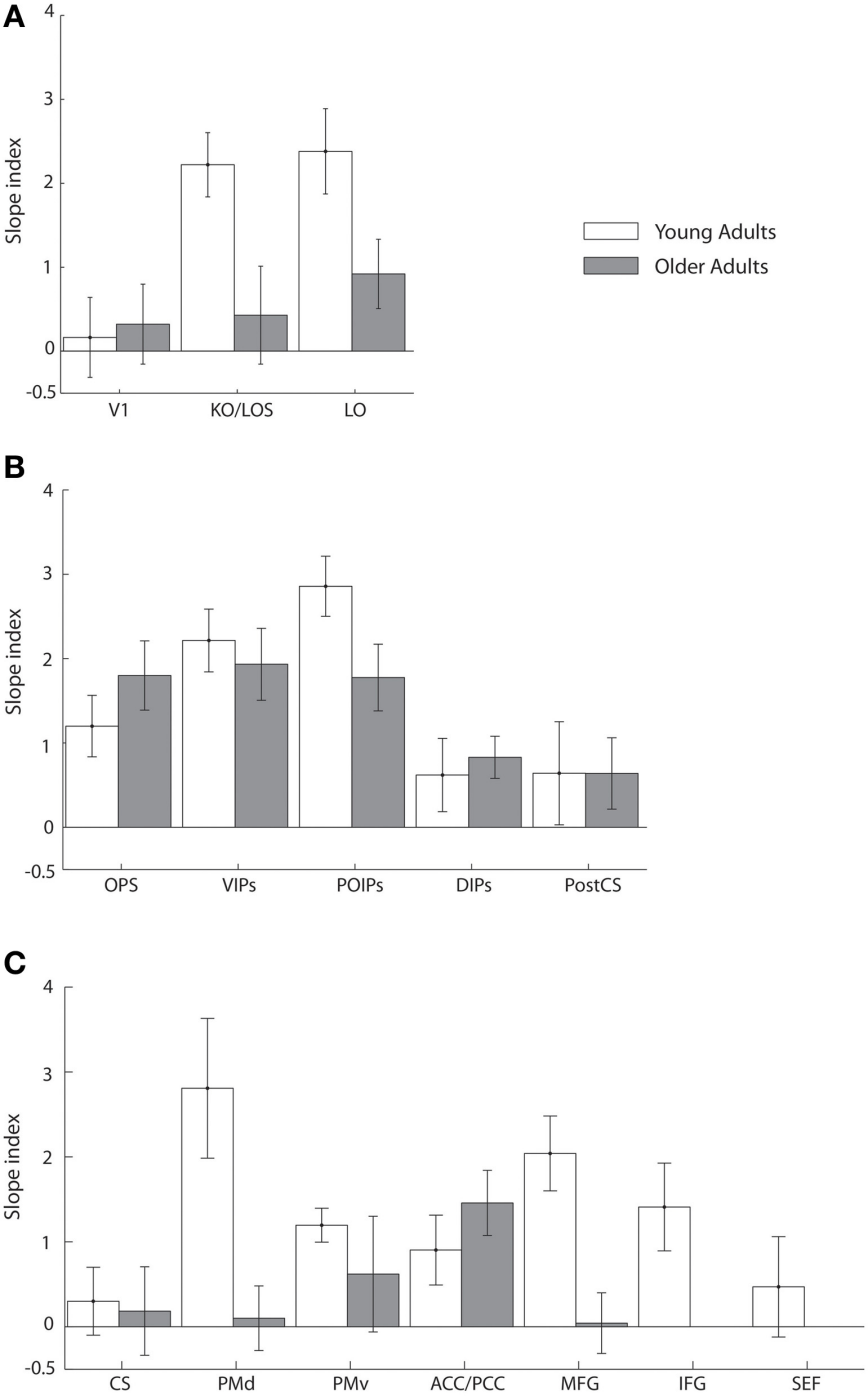
**Slope index for fMR-metric functions: main experiment.** Slope index (difference in the slope of the fMR-metric functions between pre- and post-training sessions) is plotted for young (white) and older adults (gray) performing the visual discrimination task. ROIs are grouped together by lobe, **(A)** occipital **(B)** parietal **(C)** frontal. Error bars indicate the 95% confidence interval calculated using a bootstrap procedure.

To quantify learning-dependent changes in these regions, we computed the difference in the slope of the fMR-metric functions between the pre- and post-training sessions for each participant (Figure 5). This slope index was calculated only for ROIs with significantly fitted fMR-metric functions (non-significantly fitted functions were observed in the insula for young and the insula and precuneus for older adults; see also **Table S2** for significance of the fitting for each ROI). A bootstrap analysis (2000 iterations) was used to calculate the 95% confidence intervals for the slope index. This analysis showed learning-dependent changes in young adults in an extended network of higher occiptotemporal, parietal, and frontal regions. However, for older adults learning-dependent changes were primarily observed in parietal regions. A Three-Way ANOVA (Greenhouse-Geisser corrected) on the slope of the fMR-metric functions before and after training showed a significant main effect of session [*F*_(1, 9)_ = 32, *p* < 0.0001], age [*F*_(1, 9)_ = 12, *p* < 0.01], and ROI [*F*_(1, 9)_ = 8.5, *p* < 0.01]. In addition we observed a significant three-way interaction between session, age and ROI [*F*_(1, 9)_ = 5.5, *p* < 0.05]. In particular, after training the slopes of the fMR-metric functions were steeper in young than older participants in occipitotemporal [*F*_(1, 9)_ = 7.13, *p* < 0.05], and frontal [*F*_(1, 9)_ = 7.59, *p* < 0.05] regions, while in parietal regions no significant differences were observed between age groups [*F*_(1, 9)_ = 0.4, *p* = 0.52]. In contrast, for V1 or CS no significant differences were observed between sessions [V1: *F*_(1, 9)_ = 0.51, *p* = 0.49; CS: *F*_(1, 9)_ = 0.29, *p* = 0.59] or age groups [V1: *F*_(1, 9)_ = 0.43, *p* = 0.52; CS: *F*_(1, 9)_ = 0.19, *p* = 0.66].

Taken together these results suggest that for young adults information about visual shape categories is shaped by learning in a network of occipitotemporal, parietal regions, and frontal regions that is known to be involved in perceptual decisions (Newsome et al., 1989; Kim and Shadlen, 1999; Shadlen and Newsome, 2001; Heekeren et al., 2004, 2006). In particular, information in these regions allows us to reliably decode the observer's decision and reflects learning-dependent changes in neural representations that relate to our ability to improve in the discrimination of visual shapes in noise with training. In contrast, learning-dependent changes in older adults are more prominent in parietal regions than occipitotemporal or frontal circuits. This finding is consistent with the role of the parietal cortex in the detection of salient stimuli in clutter (Gottlieb et al., 1998; Corbetta and Shulman, 2002; Mevorach et al., 2010) and may suggest that attentionally-guided learning—known to engage parietal circuits—may play a stronger role in older age. Finally, the lack of learning-dependent changes for both young and older adults in V1 is consistent with the role of primary visual cortex in representing the physical stimulus space rather than global form patterns. Similarly, the lack of learning-dependent changes for both young and older adults in CS is consistent with the role of primary motor cortex in motor responses and suggests that the learning-dependent changes in the fMR-metric functions for prefrontal and higher occipitotemporal areas could not be simply due to differences in motor responses between the two sessions (before vs. after training).

### Control experiment: task-related learning changes

Next, we investigated whether learning-related changes in young and older adult brains depend on the task performed by the observers during scanning. In particular, we trained a separate cohort of young and older adults following the same procedure as in the main experiment. However, during scanning these participants performed an orthogonal letter detection task (i.e., observers pressed a button when a target letter appeared) instead of the visual form discrimination task. The young observers' performance in the letter detection task ranged from 62.4 to 87.8% correct for response times (RT) between 872 ms (mean RT) and 1000 ms from stimulus onset. The older observers' performance in the letter detection task ranged from 65.9 to 84.2% correct for RT between 904 ms (mean RT) and 1000 ms from stimulus onset. These results ensure that the observers engaged fully with the task. Training resulted in improved perceptual sensitivity in both young and older adults similar to that observed in the main experiment. Two-Way ANOVA (session × age) on the slope of the psychometric functions showed a significant main effect of session [*F*_(1, 14)_ = 28, *p* = 0.007], supporting training-dependent improvement in task performance. No significant main effect of age [*F*_(1, 14)_ = 1.006, *p* = 0.34], or an interaction [*F*_(1, 14)_ = 0.14, *p* = 0.72] were observed. No significant differences in slope were observed between young and older adults before [*t*_(1, 7)_ = 0.2, *p* = 0.8] or after [*t*_(1, 7)_ = 0.41, *p* = 0.6] training.

**Figure 6:**
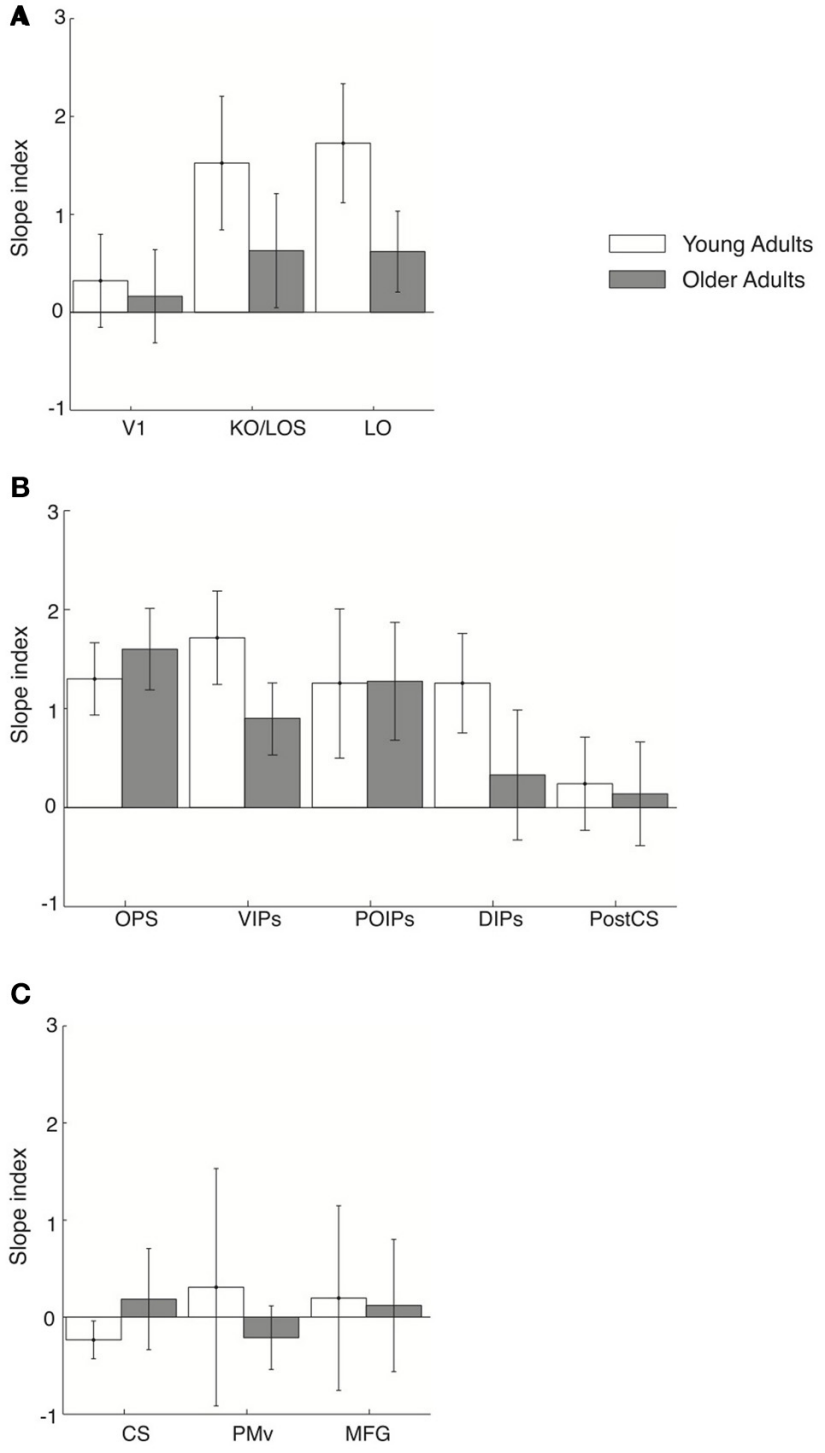
**Slope index for fMR-metric functions: control experiment.** Slope index (difference in the slope of the fMR-metric functions between pre- and post-training sessions) is plotted for young (white) and older adults (gray) performing the control (target letter detection) task. ROIs are grouped together by lobe, **(A)** occipital **(B)** parietal **(C)** frontal. Error bars indicate the 95% confidence interval calculated using a bootstrap procedure.

We then investigated learning-dependent changes when observers performed this control task by comparing the slopes of fMR-metric functions before and after training in the same ROIs used in the analysis of the main experiment (Figure 6). For young adults, fMR-metric functions showed significantly steeper slopes after than before training in KO/LOS [*F*_(1, 7)_ = 5.8, *p* = 0.033], LO [*F*_(1, 7)_ = 7, *p* = 0.025], VIPs [*F*_(1, 7)_ = 6.2, *p* = 0.03], and DIPs [*F*_(1, 7)_ = 7.8, *p* = 0.02], consistent with improvement in perceptual sensitivity after training. However, for older adults, fMR-metric functions showed significantly steeper slopes after than before training mainly in posterior parietal regions: OPS [*F*_(1, 7)_ = 9.3, *p* = 0.008], VIPs [*F*_(1, 7)_ = 7.4, *p* = 0.022], and POIPs [*F*_(1, 7)_ = 6.3, *p* = 0.032]. However, no significant differences were observed in the slopes of fMR-metric functions before and after training for frontal regions {PMv [*F*_(1, 7)_ = 0.145, *p* = 0.71], MFG [*F*_(1, 7)_ = 1.6, *p* = 0.32]} in either young or older adults (note that classification accuracies across signal levels were not significantly fit in most of the frontal regions: PMd, ACC/PCC, IFG, SEF). Finally, as in the main experiment, the slopes of the fMR-metric functions were not significantly different before and after training in primary visual or motor regions {V1 [*F*_(1, 7)_ = 0.47, *p* = 0.51], CS [*F*_(1, 7)_ = 0.09, *p* = 0.91]}. To directly compare between the main and control experiment, we conducted a Two-Way ANOVA [factors: session (pre-, post-training) × experiment (main, control)] for each ROI and age group. Significant differences in slope index were observed only for frontal regions in young adults; that is, we observed a significant effect of experiment [PMv: *F*_(1, 14)_ = 5.53, *p* = 0.038; MFG: *F*_(1, 14)_ = 6.55, *p* = 0.02] and a significant interaction between experiment and session [PMv: *F*_(1, 14)_ = 7.18, *p* = 0.016; MFG: *F*_(1, 14)_ = 12.7, *p* = 0.003].

These results suggest that higher occipitotemporal regions in young adults and posterior parietal regions in both young and older adults contain information related to the learned discrimination of global visual forms from background noise even when observers are not actively performing the discrimination task. However, frontal circuits only show learning-dependent changes that reflect the observers' behavioral choice when observers are engaged in visual form discrimination, consistent with the role of these regions in perceptual decision making (Newsome et al., 1989; Kim and Shadlen, 1999; Shadlen and Newsome, 2001; Heekeren et al., 2004, 2006). Thus, learning in higher occipitotemporal and posterior parietal regions shapes the representation of perceived visual categories that are sustained independent of the task, whereas task-related changes in frontal areas may reflect changes in perceptual sensitivity in context of the visual discrimination task.

### Control analyses

Our results show that dissociable circuits in young and older adults support learning-dependent improvement in perceptual sensitivity for the discrimination of visual forms. Here, we summarize the results of additional analyses that we conducted in order to control for alternative explanations of our findings.

First, the fMR-metric functions for both young and older adults were derived from training and testing the SVM across stimulus conditions. In this control analysis (**Figure S1**), for each ROI we trained the SVM on trials from the 100% signal condition and tested the classifier's accuracy in discriminating concentric vs. radial stimuli from data across all stimulus conditions using an independent dataset. For each observer, we calculated the mean performance of the classifier (proportion of trials correctly classified for each stimulus condition) across cross-validations. For each session, classifier performance was averaged across observers and fitted using a cumulative Gaussian. Qualitatively, the fMR-metric curves (**Figure S1**) are very similar to those shown in Figures 3, 4, demonstrating that the representation of learning effects in these regions does not depend on the classification method used.

Second, to control for the possibility that these results are due to random correlations in the data, we computed the fMR-metric functions from randomly permuted fMRI patterns (i.e., we randomized the correspondence between fMRI data and stimulus labels and estimated the classifier prediction for each stimulus condition). The lack of significant fits (**Figure S2**) suggests that the MVPA predictions could not be simply accounted for by random variations in the data but rather reflect a link between task-relevant behavioral performance and activation patterns.

Third, both young and older adults performed the same visual discrimination task during scanning to ensure that fMRI analyses were not confounded by differences in task performance. Young adult performance ranged from 56.3 to 85.2% correct for RT between 832 ms (mean RT) and 1500 ms from onset of the response cue, while for older adults from 52.4 to 83.3% correct for RT between 859 ms (mean RT) and 1500 ms. Task performance was not significantly different between age groups [accuracy: *F*_(1, 18)_ = 1, *p* = 0.3; RT: *F*_(1, 18)_ = 0.1, *p* = 0.8] suggesting that both young and older adults engaged fully in the task. Thus, the differences in MVPA activation patterns that we observed between young and old adults cannot be explained by differences in task difficulty between age groups or a general slowing of cognitive processing in older adults (Kosnik et al., 1988; Porciatti et al., 1999).

Fourth, the cued-delay paradigm we used controlled for differences in the observers' RT. That is, observers made their decision during the delay after stimulus offset and waited for the cue before they could select the correct motor response, resulting in similar RT across stimulus conditions. As the stimulus-response association was randomized across trials, the motor response could not be anticipated on a given trial. As an additional control, for both young and older adults we used the searchlight approach to search for brain patterns that contained reliable information with which to classify the finger (i.e., button press) used by the observers to indicate their behavioral choice. We performed this analysis separately using the first and second volume of each trial (**Figure S3**). No significant accuracies for this classification were observed in occipitotemporal, intraparietal, or frontal regions of either young or older adults, suggesting that results in these areas cannot be simply explained on the basis of motor responses. Although these analyses and our experimental design (i.e., two separate fMRI volumes for stimulus presentation vs. motor response in each trial) allow us to rule out confounds related to motor responses, it is still not possible to separate fMRI signals related to the stimulus from signals related to the decision process due to the limited temporal resolution of fMRI. As shown in our previous work, simultaneous EEG-fMRI recordings allow us to discriminate these processes and identify the spatio-temporal brain patterns that may support visual form learning (Mayhew et al., 2012).

Fifth, differences in activation patterns observed between young and old adults need to be interpreted with caution due to the possibility that they originate from age-related changes in vascular reactivity rather than differences in underlying neuronal activity (D'Esposito et al., 1999, 2003; Restom et al., 2007). To control for this possibility, we acquired fMRI data in both young and old adults during a 10-s breath-holding task (Handwerker et al., 2007). The BOLD signal change induced by the hypercapnic challenge of this task was used as an estimate of the vascular reactivity in every voxel. For each observer we used a GLM analysis (*p* < 0.05 uncorrected) to identify the voxels in each ROI that displayed a significant change in BOLD signal in response to the breath-holding task. On average across all ROIs the proportion of voxels selected was 96% (±2%) in young adults and 95% (±3%) in older adults. For every selected voxel, we averaged the BOLD time course across breath-hold trials and calculated the mean percentage BOLD signal change relative to the mean of the two pre-stimulus time points. We used the BOLD response amplitude to the breath-holding task to normalize the stimulus evoked BOLD signal for each observer, as previously described (Handwerker et al., 2007). For each voxel, we divided the percent BOLD signal evoked by the experimental task by the percent BOLD evoked by the hypercapnic breath-holding task. We then used the normalized signal time course for the multi-voxel pattern classification and the fMR-metric functions (Figures 3, 4; **Figures S1, S2**). **Figure S4A** demonstrates that the amplitude of the BOLD response to breath-holding was significantly [*F*_(1, 24)_ = 12, *p* < 0.01] reduced in older compared to young adults across ROIs, consistent with previous studies (Handwerker et al., 2007). However, we found no significant effect of ROI [*F*_(1, 24)_ = 1, *p* = 0.4] or interaction between ROI and age group [*F*_(1, 24)_ = 0.23, *p* = 0.64] suggesting that differences in vascular reactivity between age groups cannot explain the differences in MVPA searchlight activation patterns observed between young and older adults (i.e., the lack of significant activation in the frontal brain areas of older adults).

Sixth, previous neuroimaging studies have reported that older adults show decreases in the spatial extent of activation (D'Esposito et al., 1999; Buckner et al., 2000; Huettel et al., 2001) and longer latencies of the peak BOLD hemodynamic response (HR) compared to young adults (Taoka et al., 1998; Huettel et al., 2001). To control for such differences, we measured the HR to an 8 Hz reversing checkerboard stimulus in all ROIs, for each observer. The mean percent BOLD signal change to the checkerboard stimulus is shown for each ROI in **Figure S4B**. No significant difference in the peak HR latency [*F*_(1, 24)_ = 0.43, *p* = 0.52] or amplitude [*F*_(1, 24)_ = 2.1, *p* = 0.15] were observed between young and older adults. The lack of a significant interaction between ROI and age group [*F*_(1, 24)_ = 0.8, *p* = 0.46] justified the use of the same hemodynamic lag (4 s) for both young and older adults in the voxel time series used in the MVPA. This result was corroborated by analysis of the functional signal to noise ratio (fSNR, calculated as the mean signal change in response to the main experiment task across voxels), for each ROI used for the MVPA (**Figure S4C**). This analysis showed no significant difference in fSNR across ROIs, [*F*_(1, 24)_ = 2.2, *p* = 0.12], age groups [*F*_(1, 24)_ = 1.2, *p* = 0.36] and no interaction between ROI and age group [*F*_(1, 24)_ = 0.55, *p* = 0.65]. Taken together, these analyses provide evidence that the differences in MVPA searchlight activation patterns between young and older adults could not simply be accounted for by differences in the HR (peak latency or amplitude) between the groups.

Further, null-results in fMRI studies need to be explained with caution, as they could be due to methodological limitations. Lack of differences in brain patterns between age groups could be ascribed to either intrinsic insufficient sensitivity of fMRI data in these regions, or a true underlying lack of difference between the neuronal information carried by these areas. However, our analysis of functional SNR demonstrates that we recorded with similar sensitivity in young and older adults across areas, allowing us to compare between brain regions and age groups.

Finally, eye movement recordings conducted during scanning showed that it is unlikely that learning-dependent changes in fMRI activations patterns were significantly confounded by eye movements. That is, no significant differences in the eye position, the number and amplitude of saccades across stimulus conditions and recording sessions were observed in either young or older adults (**Figures S5A, S5B**).

## Discussion

Our findings demonstrate the learning enhances perceptual sensitivity in the discrimination of visual shapes in both young and older adults, suggesting that the ability for visual form learning is maintained in older age. However, we show that the neural circuits that mediate this ability for visual form learning across the lifespan differ with age despite similar learning-dependent improvements across age groups.

Our work advances our understanding of the neural mechanisms that mediate visual learning in older age in two main respects. First, previous studies have shown that the ability to integrate contours in clutter (Del Viva and Agostini, 2007; Roudaia et al., 2008) and suppress the background (Betts et al., 2005, 2009) deteriorates in older age. It is possible that this is due to weakening of inhibitory processes (Leventhal et al., 2003; Hua et al., 2010) or attentional functions in aging (Ball et al., 1990; Kane et al., 1994). However, our results provide evidence that learning enhances visual form discrimination in clutter for both young and older adults. This is consistent with previous studies showing that learning to discriminate visual features (i.e., texture, motion) in young and older adults (Andersen et al., 2010; Bower and Andersen, 2011) may enhance performance efficiency (Gold et al., 2004), improve exclusion of external noise and reduce internal noise (Dosher and Lu, 1999). Although our experimental paradigm does not allow us to discriminate the effect of internal vs. external noise on learning performance in ageing, our results provide evidence that learning alters not only local feature processing but also global form perception in aging.

Second, our fMRI results demonstrate that visual form learning in older age engages parietal regions, suggesting that learning may relate to the enhancement of parietal attentional functions that mediate our ability to suppress irrelevant information and enhance the salience of behaviorally relevant targets in cluttered scenes (Gottlieb et al., 1998; Corbetta and Shulman, 2002; Mevorach et al., 2010). In contrast, for young adults visual shape learning engages an extended network of occipitotemporal, parietal, and frontal regions that is known to be involved in perceptual decisions (Newsome et al., 1989; Kim and Shadlen, 1999; Shadlen and Newsome, 2001; Heekeren et al., 2004, 2006). This finding is consistent with our previous studies showing that this network of areas is also involved in visual form categorization and mediates learning-dependent changes in the observers' choice (Li et al., 2009; Mayhew et al., 2012).

Further, the reduced contribution of frontal circuits in visual form learning in older adults is consistent with our previous studies on category learning (Mayhew et al., 2010) and could not be simply attributed to differences in task difficulty between age groups, as task performance was similar for young and older observers. Is it possible that differences in the response procedure for young and older participants may account for the differences observed in frontal activation patterns between age groups? In particular, the task design may have placed higher demands on the memory of young adults that were required to switch stimulus-response mapping based on a cue, compared to older adults that simply had to delay their response. However, young participants had thoroughly practiced switching responses prior to the scanning sessions, ensuring high performance in this task. Further, this memory load was consistent before and after training for both young and older adults (i.e., the same task was used in both sessions), therefore it cannot explain learning-dependent changes in fMRI signals observed in frontal brain regions. Understanding age-related changes in frontal cortex remains a challenge as some studies show gray and white matter loss (Resnick et al., 2003; Bartzokis et al., 2004; Head et al., 2004; Madden et al., 2004; Salat et al., 2004) and functional underactivation (Grady et al., 1995; Moscovitch and Winocur, 1995; West, 1996; Cabeza et al., 1997; Rypma and D'Esposito, 2000; Rypma et al., 2001; Logan et al., 2002), while others demonstrate hyperactivation potentially related to compensatory mechanisms that may support brain plasticity in older age (Heuninckx et al., 2008; Reuter-Lorenz and Cappell, 2008; Berchicci et al., 2012; Fakhri et al., 2012).

Third, our results show that testing observers in a control task rather than a visual from discrimination task results in learning-dependent changes in fMRI activation patterns in higher occipitotemporal and posterior parietal regions, but not frontal circuits. Thus, learning may modulate read-out signals in posterior regions related to global form representations independent of the task, whereas task-dependent frontal activations may reflect changes in sensitivity with training in the context of perceptual decision making. This finding is consistent with our previous work on category learning (Li et al., 2009) and the proposed role of frontal areas in adaptive coding for complex cognitive tasks (Miller, 2000; Duncan, 2001; Koechlin and Summerfield, 2007). It is unlikely that the learning induced changes we observed in occipitotemporal areas resulted from learning specific stimulus exemplars, as the stimuli tested during scanning differed in their visual properties (i.e., signal level) from the stimuli presented during training. Further, we controlled for the possibility that the results could be due to memorized stimulus-response associations by randomizing the motor responses based on the cue, and introducing a task requiring a motor response orthogonal to the stimulus categories in the control experiment.

Finally, our work introduces a new methodological approach to the study of the aging brain by comparing the information content rather than the overall signal amplitude of brain regions in young and older adults. Comparing fMR-metric functions that reflect the choices of an MVPA classifier to psychometric functions that reflect the observers' choices, we discern cortical areas where visual form representations change with learning according to the observers' behavior. These learning-dependent activity changes may reflect changes in the selectivity of single neurons, correlations across local neural populations, or input from local or distant neural circuits. Discerning these mechanisms with fMRI alone is not possible due to the limited resolution of the technique and the complex nature of the BOLD signal. Therefore, future work combining fMRI and electrophysiology signals is necessary to shed more light into the neural mechanisms that mediate visual form learning across the lifespan.

### Conflict of interest statement

The authors declare that the research was conducted in the absence of any commercial or financial relationships that could be construed as a potential conflict of interest.
